# Use of Botulinum Toxin in Spasmodic Dysphonia: A Review of Recent Studies

**DOI:** 10.7759/cureus.33486

**Published:** 2023-01-07

**Authors:** Hammaad A Khan

**Affiliations:** 1 Otolaryngology - Head and Neck Surgery, Royal Lancaster Infirmary, Lancaster, GBR

**Keywords:** vocal fold injection, laryngeal dystonia, vocal tremors, chemodenervation, spasmodic dysphonia, botulinum toxin

## Abstract

Spasmodic dysphonia (SD), also known as laryngeal dystonia, is a neurological voice disorder that causes involuntary spasms of the vocal cord muscles. This impacts speech to varying degrees and results in strained and strangled voice quality, as in adductor spasmodic dysphonia, or weak, quiet, and breathy, as in abductor spasmodic dysphonia. While there is currently no cure for SD, voice therapy and chemodenervation with botulinum toxin (btx) injections remain the mainstay of management. Surgery may be performed in some cases; however, btx injections are widely used to treat both adductor and abductor forms of SD. While btx injections may show vocal improvement in both types of SD, results can depend on several factors such as the general health of the patient, onset and severity of the condition, dosage, interval between injections, and expertise of the practitioner. While many studies have documented the efficacy of btx for improving vocal symptoms in individuals with SD, this review aims to discuss some of those studies from the last 10 years.

## Introduction and background

Spasmodic dysphonia (SD), or laryngeal dystonia, is a neurologic condition that affects voice and speech and mostly appears in middle age. SD is usually sporadic and affects intrinsic laryngeal muscle control only occurring during phonation [1].

SD is a lifelong condition and is not curable which causes involuntary spasms of the vocal cords through hyperadduction and hyperabduction [2]. As a result, the individual’s voice can be affected, and the severity can range from difficulty in saying a word or two to being unable to talk at all. In some cases, this may be temporary, but where this persists, it can be improved through treatment.

There are three types of SD: the most prevalent form of SD is adductor spasmodic dysphonia (AdSD), followed by abductor spasmodic dysphonia (AbSD), and the mixed type [1].

AdSD is characterized by hyperadduction, the most common type of SD, and constitutes about 85-90% of cases. Sudden involuntary spasms trigger the vocal cords which become stiff and slam shut. These spasms interfere with the vibration of the vocal cords and produce a strained voice despite the patient making a full effort. Spasms do not occur when the person is whispering, laughing, singing, speaking at a high pitch, or speaking while inhaling. In addition, it has been noted that stress can exacerbate spasms [2].

AbSD is a less common type associated with hyperactivity of the posterior cricoarytenoid or the failure of the lateral cricoarytenoid or thyroarytenoid muscles to contract [1]. Thus, speech is interrupted, and voice quality appears strained or strangled. Speech sounds are weak, quiet, and breathy in nature. These spasms, however, are absent when the person is laughing or singing [3].

Mixed SD is very rare and is a mix of symptoms of both types of dysphonia.

Treatment

Currently, there is no cure for SD. However, the use of botulinum toxin (btx) injection remains the gold standard for the management of vocal symptoms. There are, however, certain limitations of chemodenervation with btx in managing SD. One such limitation is that btx needs to be reinjected periodically. It can be followed by a brief period of vocal instability where the voice quality is not at a desired optimum level. In addition, the results of btx injection are often not uniform and the effects may wane due to the possible formation of btx antibodies [4].

Although voice/speech therapy is also used in patients with SD, it does not result in a marked improvement on its own. However, it can be useful when administered in addition to btx injections [5]. Apart from voice therapy, SD was also historically treated with psychotherapy, but both interventions have significantly limited improvement of the condition [6].

While surgical treatment has been in place much before chemodenervation became common, its long-term efficacy has not been well-established [5].

Chemodenervation with btx continues to remain the current standard of treatment for SD, but it is important to note that suitable dosage is determined individually after a titration period. This in many cases can take months to years before symptoms are relieved. In addition, factors such as body mass index (BMI), and general health issues such as lack of vitality, limitation in performing daily activities, physical and emotional health problems, and pain can show a positive correlation with higher effective doses and may be useful in guiding clinicians during the titration period, especially in the AdSD subset of patients [7]. In addition, almost one-third of patients presenting with AdSD present with an associated vocal tremor. These patients may not respond fully to treatment by injecting btx into the TA muscle group. This may be the result of the involvement of multiple muscle groups in the tremor. Hence, patients with a combination AdSD and vocal tremor and who do not respond to btx injection in the thyroarytenoid muscle alone may benefit from adding btx in the interarytenoid muscle for improved voice outcomes [8].

The use of btx is supported by a large number of studies that conclude its effectiveness, especially in the uncomplicated adductor form of SD [5]. However, the author aims to examine studies published in the last 10 years that have not only documented the efficacy of btx for the improvement of vocal symptoms in individuals with SD but also discussed new developments in its management.

## Review

Methodology

The author searched PubMed, PubMed Central (PMC), ResearchGate, and Google Scholar for relevant keywords such as vocal fold injection, laryngeal dystonia, vocal tremors, chemodenervation, spasmodic dysphonia, and botulinum toxin, as well as Medical Subject Headings (MeSH) “botulinum toxin” and “spasmodic dysphonia” to identify all relevant articles that discuss the role of btx injections in the management of voice symptoms in SD. The author included studies from 2012 to December 2022. The reference lists for all studies and other review articles were examined to identify additional studies.

Inclusion criteria included full-text, English-language articles from the last 10 years focusing mainly on the efficacy of btx injections in SD. Exclusion criteria included studies on complications of btx, studies not published in the English language, abstract-only articles, and those discussing non-btx interventions (unless btx was compared).

Results

A search on PubMed, PMC, ResearchGate, and Google Scholar yielded 149 studies. These included clinical trials, meta-analyses, randomized controlled trials, reviews, and systematic reviews. Studies not directly related to the topic were omitted to leave 39 articles. Of these, only 20 had full text available, from which the author selected 14 articles that were found to be relevant to this review.

**Table 1:** Studies related to botulinum toxin injections in spasmodic dysphonia. SLAD-R = selective laryngeal adductor denervation-reinnervation; AdSD = adductor spasmodic dysphonia; btx = botulinum toxin; SD = spasmodic dysphonia; VHI = Voice Handicap Index; VL = video laryngoscopy; VS = voice spectrography; AbSD = abductor spasmodic dysphonia; HA = hyaluronic acid; ETV = essential tremor of the voice; EMG = electromyography

Authors and country	Patient group	Study type	Aim of the study	Conclusions
Mendelsohn and Berke, 2012, USA [9]	Two cohorts: Surgical cohort, 77 patients with a mean follow-up time of 7.54 ± 2.55 years. Injection cohort, 28 patients with a mean follow-up time of 46.37 ± 5.51 days	Prospective case study	The aim of the study was to compare the outcome of SLAD-R surgery for AdSD to that of btx injections	The study concluded that when indicated, the SLAD-R surgery for AdSD demonstrated outcomes which are equal to or superior to those of the current standard of btx injections
Rosow et al., 2013, USA [10]	305 patients receiving onabotulinum toxinA in the management of AdSD	Case series with chart review	The study aimed to assess the effect on voice improvement and duration of breathiness based on the initial dose of onabotulinum toxin A (btx-A) in the management of adductor SD and compare voice outcomes for initial bilaterally injected doses of 1.25 units (group A) vs. 2.5 units (group B) of btx-A	The study concluded that patients injected with 1.25 units bilaterally had a statistically significantly shorter duration of breathiness without a statistically significant difference in clinical effectiveness or voice outcomes. It is, therefore, recommended that a relatively low initial btx-A dose be used with subsequent titration to achieve improved voice outcomes
Esposito et al., 2015, Italy [11]	13 patients with AdSD were studied with VHI, VL, and VS before and after four consecutive treatments with onobotulinumtoxin-A	Prospective case study	This study aimed to assess the benefit of treatment with btx injections in SD by using objective measures such as VL and VS which are in accordance with a subjective assessment of the VHI questionnaire	This study supported that btx injections are an efficient treatment for SD with objective measurement. The study further concluded that the efficacy of recurring treatments was stable over time
Rosow et al., 2015, USA [12]	211 patients receiving btx injections for SD	Prospective case study	The aim of the study was to observe if demographic and environmental factors, such as age, gender, and smoking status, have an impact on the long-term stability of btx dosing in patients with SD	The dosage of btx injections for long-term treatment of SD has a significant propensity to remain stable over time. Factors such as age, gender, and smoking status do not appear to influence dosage stability. These findings should allow for better patient counseling regarding expectations for their long-term treatment
Bradley et al., 2017, USA [13]	113 patients with AdSD from 2003 to 2013 were identified from a clinical database. who had at least 10 injections	Retrospective chart review	This study aimed to identify the changes in the dosing of btx-A for AdSD over a prolonged period	Btx-A dosing for AdSD decreases consistently over subsequent injections after the initial two-dose titrations
Schuering et al., 2019, The Netherlands [14]	22 patients with AdSD	Retrospective case series	The study aimed to look at surgical interventions as an alternative to bxt, which is the current gold standard of therapy for AdSD	The study concluded that a surgical procedure such as endoscopic laser thyroarytenoid myoneurectomy can potentially offer more stable and long-lasting voice quality versus btx injections. However, in this study, 45% of patients showed deterioration after 12 months and needed a second procedure
Lerner et al., 2020, USA [15]	A total of 201 patients (52 males and 149 females) diagnosed with AdSD who received onabotulinum toxin A (btx-A) injections to the thyroarytenoid muscle for at least five years were included in the study	Retrospective review	The aim of the study was to determine the influence of gender on btx-A dosing for the treatment of AdSD symptoms	The data from this retrospective chart review revealed a statistically and clinically significant correlation between the female gender and higher average btx-A dose for symptom control in AdSD. Explanations for this observation are speculative and include a possible inverse relationship between optimal btx-A dose and vocal fold mass as well as possibly greater neutralizing antibody formation among female patients
Meyer et al., 2021, USA [16]	101 patients with SD, of whom 75 participated in the study	Prospective case series	To assess whether work productivity increased in patients with SD who were treated with btx injections, and had a 10% or greater improvement in productivity one month after the injection	This study concluded that patients with SD reported voice-related work productivity impairment, which significantly improved one month after treatment with btx injection. The association of SD with voice-related work productivity appeared greater in women than in men with comparable outcomes with btx treatment, but this exploratory gender-associated difference requires independent validation
Hyodo et al., 2021, Japan [17]	24 patients (22 with AdSD and 2 AbSD	Multicenter, placebo-controlled, randomized, double-blinded, parallel-group comparison/open-label clinical trial	The aim of the study was to evaluate the effectiveness and safety of btx-A injection in the treatment of patients with AdSD and AbSD	Btx injection was safe and efficacious and was considered the treatment of choice for SD and reduced the severity of voice disorders
Hirose et al., 2021, Japan [18]	22 patients with AdSD. The female-to-male ratio was 20:2, with a mean age of 40.0 ± 10.3 years, and a median duration of symptoms of 7.5 years	Placebo-controlled, randomized, double-blinded, parallel-group comparison/open-label clinical trial	This study aimed to analyze data to better understand the detailed chronological course and clinical factors that affect btx therapy in patients with AdSD	The study concluded that btx therapy was effective for AdSD based on both objective and subjective assessments
Woo, 2022, USA [19]	8 patients received 23 simultaneous HA/btx using LEMG	Prospective case study	The aim of the study was to analyze improvement in results by injecting btx and HA simultaneously in selected patients	The study concluded that btx injection along with HA may be helpful in patients with ETV and presbyphonia. It can also be considered in professional voice users with AdSD to reduce the side effects of btx
Rutt et al., 2022, USA [20]	36 patients who received laryngeal btx injections	A 14-item survey	The study explored non-biological factors that can affect patient experience during btx injections and their association with better or worse self-reported effectiveness	The study showed that non-pharmacological factors such as education before the procedure, body position, pain, and stress sensation before the btx injection may have a role in the effect of the injections on AdSD patients
Stone et al., 2022, USA [21]	319 patients with SD and/or ETV	Prospective case study	The study aimed to quantitatively compare the effectiveness of unilateral and bilateral btx injections in treating weak/breathy voice quality and dysphagia for patients with AdSD and/or ETV	A unilateral injection was useful for patients with pronounced dysphagia and extended periods of weak/breathy voice. Whereas bilateral btx injections are useful for patients seeking to extend their length of improved voice, provided the adverse side effects from btx-A are minimal. Unilateral injections are useful for patients with ETV at the outset of their course of treatment with btx-A
Kohli et al., 2022, USA [22]	16 patients with SD who underwent EMG-guided incobotulinum toxin-A injections.	Prospective open-label trial	To demonstrate an understanding of incobotulinum toxin-A efficacy in the treatment of AdSD	The study demonstrated that incobotulinum toxin-A is equally useful as first-line treatment or in secondary non-responders to btx-A

Discussion

This article reviews studies published between 2012 and 2022, that have documented the efficacy of btx injections for the improvement of vocal symptoms in SD. The use of btx in the management of vocal symptoms in SD has remained the gold standard for over four decades. Studies that were reviewed also concluded the same, and new research also takes into consideration dose titration, techniques, and btx variants for optimum outcomes.

There have been attempts to explore surgical alternatives to btx for positive voice outcomes. For example, patients undergoing selective laryngeal adductor denervation-reinnervation in AdSD may show equal or superior outcomes to those of btx, as demonstrated through patient-oriented measures such as the Voice Handicap Index [9]. While the scope of this review is limited to btx injections, this study was included to compare it with available surgical alternatives.

A relatively low initial btx-A dose can be used with subsequent titration to achieve improved voice outcomes and fewer side effects such as breathiness [10]. While it was concluded that btx injections are efficient in the management of voice outcomes in SD with objective measurements, a study further concluded that the efficacy of recurring treatments remains stable over a period of time [11].

Factors such as age, gender, and smoking status do not appear to influence dosage stability [12]. Regarding dosage, btx injection for AdSD decreases consistently over subsequent injections after the initial two-dose titrations [13].

A study concluded that surgical procedures such as endoscopic laser thyroarytenoid myoneurectomy can potentially offer more stable and long-lasting voice quality compared to btx injections. However, in the study, 45% of patients showed deterioration after 12 months and needed a second procedure, which poses the question of whether it is comparatively a viable alternative to btx injections [14].

Gender, however, seems to have some influence in determining the dosage and desired outcomes. A study [15] concluded statistically and clinically that there is a significant correlation between female gender and higher average btx dose for symptom control, specifically in AdSD. Another study examined the association of SD with voice-related work productivity and concluded that gender may play a role in the desired outcomes. The study demonstrated that after one month of btx treatment, a greater number of women showed better outcomes compared to men. However, this gender-associated difference requires independent validation [16].

Btx injections as the treatment of choice in SD continue to be safe and efficacious [17], which another study demonstrated based on both objective and subjective assessments [18]. To reduce the side effects of btx, introducing hyaluronic acid has also been discussed in a study [19].

Recent studies also demonstrate that non-pharmacological factors such as education before the procedure, body position, pain, and stress sensation before btx may play a role in the effect of btx on AdSD patients [20]. In addition, a study compared unilateral and bilateral btx injections quantitatively and concluded that unilateral btx is useful for patients with an extended period with weak/breathy voice quality and those with dysphagia [21].

A study also demonstrated that incobotulinimtoxinA is equally useful as first-line treatment or in secondary non-responders to onabotulinumtoxinA [22].

## Conclusions

The studies in this review have documented that btx injections continue to be the gold standard for the management of patients with SD. It is evident that globally, clinicians who have been exploring the best possible voice outcomes in patients with SD have confidence in the use of various variants of btx for different types of SD. This is mainly because btx injections not only provide a safe and reliable means of improving SD but are also easily available compared to other complex surgical interventions. While socioeconomic factors, patient counseling, gender, BMI, and other external factors may play some role, there is not enough evidence to conclude a strong correlation between dose variation in btx therapy and voice outcomes in SD. Therefore, more research is needed for a better understanding of these factors.
